# Comparison of the effectiveness of immersive and non-immersive virtual reality in the treatment of vertigo in patients with peripheral vestibular dysfunction: a systematic review and meta-analysis

**DOI:** 10.3389/fneur.2025.1638868

**Published:** 2025-07-17

**Authors:** Xiaoyi Liu, Sha Yang, Yiru Wang, Ziwei Tong, Xiao An, Xiaoqing Ren, Xu Sun, Zhicong Zhou, Hong Wang, Xiaoying Liu

**Affiliations:** ^1^School of Nursing, Shandong Second Medical University, Weifang, China; ^2^The First Affiliated Hospital of Shandong First Medical University, Jinan, China

**Keywords:** peripheral vestibular dysfunction, virtual reality, vertigo, systematic review, meta-analysis

## Abstract

**Background:**

Vertigo is the most common clinical manifestation in patients with peripheral vestibular dysfunction (PVD), and severe episodes may be accompanied by nystagmus, tinnitus, and hearing loss, which can seriously affect quality of life. Virtual reality (VR) technologies (immersive or non-immersive) play an important role in improving vertigo in patients with PVD, but the comparative effectiveness of VR technologies with different levels of immersion is unknown.

**Objective:**

To investigate the effectiveness of VR technology at different immersion levels in reducing vertigo symptoms in patients with PVD.

**Method:**

PubMed, MEDLINE, Embase, Cochrane Library, Web of Science, CINAHL and 4 Chinese databases were systematically searched. Standardized mean difference (SMD) was calculated using RevMan 5.4 software, and risk of bias was assessed using the Cochrane Collaboration tool and Stata software. The review process was reported according to PRISMA.

**Results:**

Twelve studies involving 600 participants met the inclusion criteria. The results indicated that both non-immersive and immersive VR significantly improved vertigo symptoms in patients with PVD; however, the immersive VR intervention demonstrated greater effectiveness (SMD = −2.08, 95% CI = −3.13 to −1.04, *p* < 0.001). Further subgroup analyses revealed that immersive VR intervention programs with a duration of ≤7 weeks (SMD = −2.73; 95% CI = −4.17 to −1.28, *p* < 0.001), a single intervention duration of <30 min/ times (SMD = −2.80, 95% CI = −4.89 to −0.70, *p* = 0.009), and a frequency of ≥5 times/week (SMD = −2.64; 95% CI = −4.91 to −0.38, *p* = 0.02) were more effective in alleviating vertigo symptoms.

**Conclusion:**

Immersive VR has been shown to be more effective in alleviating vertigo symptoms in patients with PVD. Specifically, an immersive VR program that includes an intervention period of ≤7 weeks, a single intervention duration of <30 min, and an intervention frequency of ≥5 times/week is recommended for optimal improvement of vertigo symptoms. Further high-quality, multicenter randomized controlled trials are recommended to confirm the findings of this study. Healthcare professionals should focus on the individual differences of elderly patients with PVD and provide personalized VR vestibular rehabilitation programs for optimal rehabilitation outcomes.

**Systematic review registration:**

https://www.crd.york.ac.uk/PROSPERO/, identifier CRD42025638469.

## 1 Introduction

Peripheral vestibular dysfunction (PVD) refers to a vestibular imbalance associated with lesions of the vestibular receptors and the extracranial segment of the vestibular nerve (1). Vertigo is the most prevalent clinical manifestation in patients with PVD, characterized by recurrent episodes that often remain unresolved (2, 3). Approximately 30% of adults report experiencing dizziness or vertigo, and severe episodes may be accompanied by nystagmus, tinnitus, and hearing impairment (4–6). Impaired vestibular function may lead to instability in gaze and gait, imbalance, and compromised spatial orientation, resulting in a 12-fold increase in the incidence of falls compared to healthy individuals (7, 8). A 16-year cross-sectional study reported that approximately 60,060/805,454 patients experienced falls due to symptomatic dizziness or PVD, with the incidence increasing with age; about 5.7% of these falls resulted in severe injuries or even death (9, 10).

The American Physical Therapy Association’s Clinical Practice Guidelines indicate that vestibular rehabilitation therapy (VRT) is a standard treatment for vestibular dysfunction (11). Early intervention is essential for establishing central vestibular compensatory mechanisms, which subsequently improve the patient’s vertigo symptoms (12, 13). However, conventional VRT is often perceived as monotonous, leading to poor patient compliance and suboptimal therapeutic outcomes (14, 15). Virtual reality (VR), an emerging technology that employs advanced computing, accelerates the development of vestibular compensation and adaptation mechanisms by stimulating the plasticity of the central nervous system (16, 17). Additionally, VR provides real-time simulation, interaction, and gamification features that enhance the enjoyment of vestibular training, thereby improving patients’ motivation for rehabilitation and increasing their participation and compliance (18, 19). Consequently, VR plays a significant role in alleviating vertigo symptoms in patients with PVD due to its diverse and interactive capabilities (18, 20).

Immersive and non-immersive VR applications differ primarily in the depth of experience and interaction from the patient’s perspective (21, 22). Non-immersive VR relies on accessories such as keyboards, mice, microphones, or motion sensors to facilitate interactions that translate the patient’s physical behavior into on-screen movements (23, 24). In contrast, immersive VR employs a head-mounted display along with specialized biomedical sensors to provide complete immersion and interaction with the patient’s surroundings within a virtual environment (25, 26). The integration of VR with vestibular rehabilitation enhances multisensory stimulation, including audiovisual and tactile sensations, through virtual daily activities and game design. This approach not only optimizes vestibular rehabilitation methods and increases patients’ self-efficacy but also stimulates the vestibular compensatory mechanism, significantly improving rehabilitation outcomes compared to the single-motion stimulation of conventional VRT.

Current evidence indicates that VR (both immersive and non-immersive) is superior to conventional VRT (27, 28). However, there remains a lack of clear evidence regarding which level of immersion in VR devices produces more effective rehabilitation outcomes. Additionally, further exploration is needed to determine the optimal intervention strategies, including duration and frequency of treatment. Consequently, the primary aim of this study is to compare the effectiveness of immersive VR and non-immersive VR in alleviating dizziness in patients with PVD. The secondary objective is to explore the ideal intervention scheme for the optimal VR device.

## 2 Methods

This study was conducted in accordance with the Preferred Reporting Items for Systematic Evaluation and Meta-Analysis (PRISMA) statement (29), and the methodology of this systematic review and meta-analysis has been published in Prospero Platform (CRD42025638469).

### 2.1 Search strategies

We searched PubMed, MEDLINE, Embase, Cochrane Library, Web of Science, CINAHL, CNKI, Wanfang database, VIP and CBM, and the search period was from the establishment of the database to June 2025. The search strategy used MeSH terms and keywords related to “virtual reality” and “peripheral vestibular dysfunction,” and the references of the included literature were manually searched. The following presents an example of our search process within the PubMed database (Table 1), and the complete search strategy is detailed in Supplementary materials S1.

**Table 1:** Search strategy for PubMed database.

No.	Search items
1	(((((((((((((((((peripheral vertigo[MeSH Terms]) OR (peripheral vertigo[Title/Abstract])) OR ((peripheral[Title/Abstract]) AND (vertigo[Title/Abstract]))) OR (meniere disease[MeSH Terms])) OR (Meniere’s disease[Title/Abstract])) OR (Meniere’s disease)) OR (Benign Paroxysmal Positional Vertigo)) OR (Benign Paroxysmal Positional Vertigo[Title/Abstract])) OR (Vestibular Neuritis[Title/Abstract])) OR (Vestibular Neuritis)) OR (Vestibular Neuritis[MeSH Terms])) OR (BPPV[Title/Abstract])) OR (BPPV)) OR (Peripheral vestibular dysfunction)) OR (Peripheral vestibular dysfunction[Title/Abstract])) OR (Peripheral vestibular lesion[Title/Abstract])) OR (vertigo*[Title/Abstract]))
2	((((((((((((virtual reality[MeSH Terms]) OR ((virtual[Title/Abstract]) AND (reality[Title/Abstract]))) OR (virtual reality[Title/Abstract])) OR (virtual environment[Title/Abstract])) OR (digital technology[Title/Abstract])) OR (video game[Title/Abstract])) OR (exergaming[Title/Abstract])) OR (virtual rehabilitation[Title/Abstract])) OR (VR[Title/Abstract])) OR (Three-dimensional[Title/Abstract])) OR (3D Virtual Reality[Title/Abstract])))
3	#1 AND #2

### 2.2 Eligibility criteria

Inclusion criteria:

(1) Participants: patients aged ≥18 years with a diagnosis of PVD and concomitant vertigo were included in the study.
(2) Intervention: VR (immersive or non-immersive).
(3) Comparison: conventional VRT and physiotherapy.
(4) Outcomes: Vertigo symptoms were assessed using the Visual Analog Scale (VAS), Dizziness Handicap Inventory (DHI), Vertigo Symptom Scale (VSS), and Vertigo Symptom Scale-Short Form (VSS-SF).
(5) Research designed as a randomized controlled trial (RCT) has no restrictions on the frequency, intensity, and duration of the intervention.

Exclusion criteria:

(1) Patients in the midst of an acute vertigo attack.
(2) Studies with incomplete data or where conversion is not possible.
(3) Studies not in English or Chinese.
(4) Studies without full text.

### 2.3 Literature screening

Literature screening was performed in EndNote. Two reviewers (XYL and SY) independently screened each paper according to the inclusion and exclusion criteria, first excluding duplicate studies, then independently screening the full text based on title and abstract, and finally cross-checking. In case of disagreement, a third reviewer (YRW) was consulted.

### 2.4 Data extraction and quality assessment

Two reviewers utilized an Excel sheet to extracted data after reading the full text. The data extracted included the following variables: first author, year of publication, sample size, age, intervention and control group, intervention period, single intervention duration and intervention frequency, level of immersion, type of VR (equipment, VR environment and software), and outcome indicators. Use a separate form (Supplementary material S2) to record the raw outcome data reported for each study. The quality of RCTs was assessed using the Cochrane Risk of Bias tool (30), which includes method of randomization, allocation concealment, blinding of study subjects and investigators, blinding of assessors, completeness of outcome indicators, selective reporting status, and other biases. Each item was categorized as “low risk,” “unclear,” or “high risk.” In case of disagreement regarding the results, this was done by mutual agreement or by consulting a third reviewer.

### 2.5 Statistical analyses

Statistical analysis was performed using RevMan 5.4, and the included outcome indicators were continuous variables, so mean difference (MD) or standardized mean difference (SMD) was used as the effect indicator, and 95% CI was the effect analysis statistic. Heterogeneity was analyzed using the *χ^2^* test and the *I^2^* statistic, if there was no heterogeneity among the study results (*p* > 0.1, *I^2^* < 50%), the fixed-effects model was used for the analysis; if there was heterogeneity (*p* ≤ 0.1, *I^2^* ≥ 50%), the random-effects model was used, and sensitivity analysis was performed. The stability of the results was tested by excluding literature on a case-by-case basis to identify sources of heterogeneity. We performed subgroup analysis according to different immersion levels. And further subgroup analyses of the optimal immersion methods were performed to further identify the sources of heterogeneity. Egger’s test was performed using Stata18 software. *p* < 0.05 indicates a statistically significant difference. Some studies did not provide efficacy data regarding the improvement of dizziness symptoms; therefore, we utilized the data transformation formula recommended by Chi (31) and Luo (32) to compute the mean and standard deviation, which were obtained by XYL under the supervision of another reviewer (YRW).

## 3 Results

### 3.1 Search results

**Figure 1:**
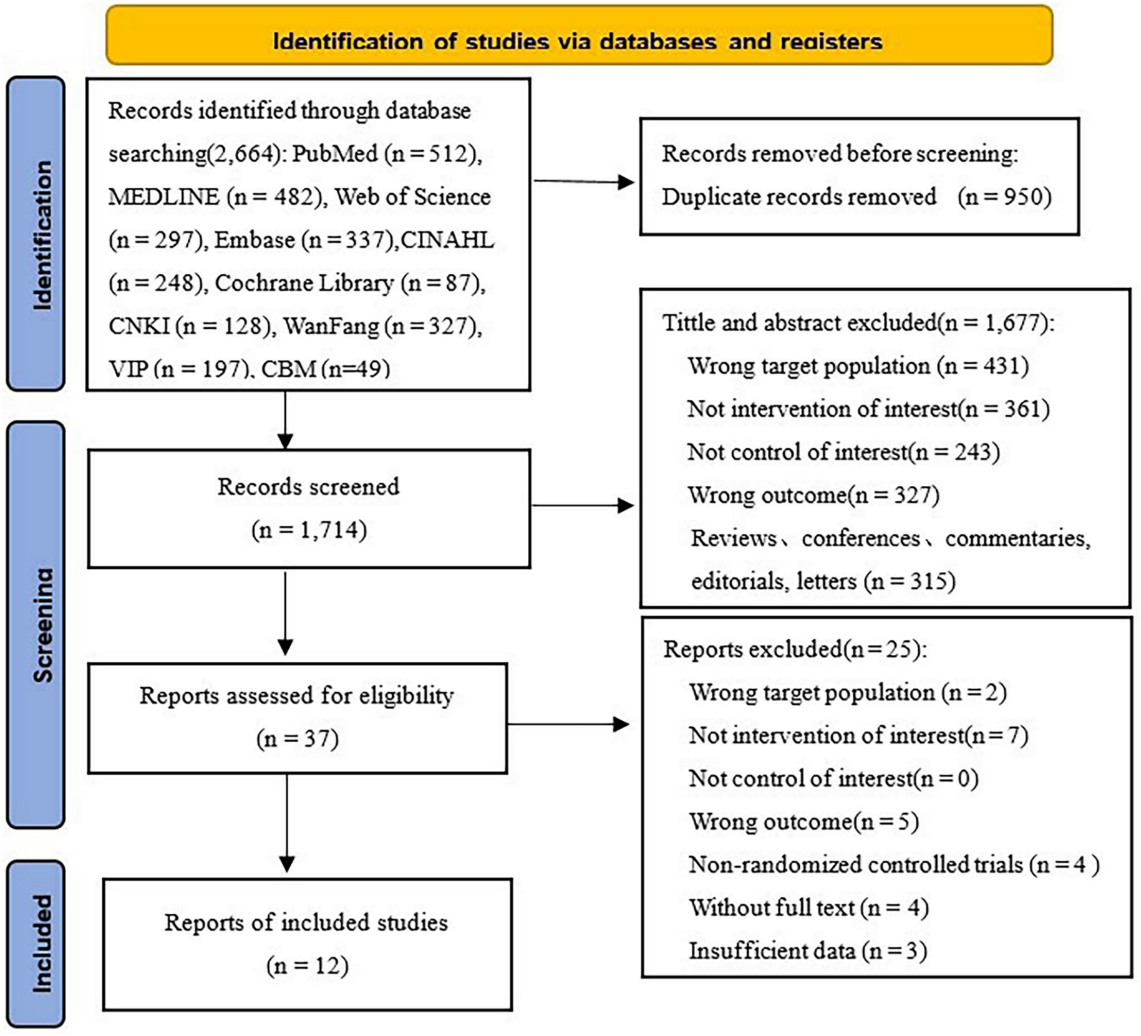
PRISMA flow diagram of study selection.

An initial search identified a total of 2,664 academic articles, including 1,963 in English and 701 in Chinese. Utilizing EndNote X9.1, 950 redundant entries were eliminated, resulting in a total of 1,714 articles. Following a review of the titles and abstracts, 1,677 articles were excluded for several reasons: incorrect target populations, lack of intervention of interest, absence of appropriate controls, wrong outcomes, etc. After a thorough review of the literature, we excluded 25 studies for the same reasons (see Figure 1 for further details). Ultimately, a total of 12 RCTs (33–44) were finally included.

### 3.2 Study characteristics

Table 2 outlines the detailed characteristics of each study included in our analysis. Among the twelve studies examined, the majority were conducted in Turkey (40–42) (three studies), followed by Brazil (33, 39), Italy (34, 36), and China (38, 43) (two studies), with the UK (35), Poland (37), and Jordan (44) (one study). The publication dates spanned from 2011 to 2025; eight of them after 2020 (37–44). A total of 600 participants were included in the meta-analysis, comprising 304 in the intervention group and 296 in the control group. The average age of participants ranged from 39.6 ± 8.75 to 70 ± 6 years. Regarding PVD types, four studies focused on unilateral vestibular hypofunction (34, 36, 37, 41), three examined benign paroxysmal positional vertigo (BPPV) (38, 42, 43)[with one specifically addressing residual symptoms of BPPV (43)], and two investigated elderly patients with dizziness (39, 40). Additionally, one study each explored persistent postural-perceptual dizziness (PPPD) (44), adults with dizziness (35), and Ménière’s disease (33).

**Table 2:** Characteristics of included studies.

Study	Age (I/C)	Participants	Intervention/VR Group	Control/comparison group	Type of VR equipment	Environment software	Outcome measures
Garcia et al. (33) Brazil	47.65/47.90	44 patients Ménière’s disease.	*N* = 23 Head-mounted display (45 min/times, 2 times/week for 6 weeks) + Light diet + 48 mg per day of betahistine.	*N* = 21 Light diet + 48 mg/day of betahistine.	Immersive head-mounted display. Balance Rehabilitation Unit (BRUTM): A computer with a test program, a 40 × 40 cm force platform, virtual reality goggles, a foam cushion.	Undescribed.	Vertigo: Dizziness Handicap Inventory (DHI).
Micarelli et al. (34) Italy	49.72 ± 10.34/50.48 ± 9.12	47 patients Unilateral vestibular hypofunction.	*N* = 23 Head-mounted display + Conventional VRT. 30–45 min/times, 2 times/week for 4 weeks.	*N* = 24 Conventional VRT. 30–45 min/times, 2 times/week for 4 weeks.	Immersive head-mounted display. HMD ‘Revelation’ 3D VR Headset (length × width × depth = 140 × 105 × 64 mm, weight = 165 g, 110° diagonal field of view) and a Windows Phone (Lumia 930, Windows 10 Mobile, Microsoft Corporation, Redmond, Washington, USA).	Track Speed Racing 3D game: Tilting the head left or right maneuvers the car to avoid veering off the road and achieve all goals before completing a lap. (Induces visual-vestibular conflict by simulating eye-head movements).	Vertigo: Dizziness Handicap Inventory (DHI).
Phillips et al. (35) UK	48.00 ± 15.00/47.00 ± 16.00	40 patients Adults with dizziness.	*N* = 10 Non-immersive gaming console. 30 min/times, 2 times/day for 16 weeks.	*N* = 11 Conventional VRT. 30 min/times, 2 times/day for 16 weeks.	Non-immersive gaming console: Wii Fit balance platform.	Balance games: Heading, Ski Jump, Ski Slalom, Tightrope, Irritating Maze, Penguin Game, Snowboarding, and Meditation.	Vertigo: Dizziness Handicap Inventory (DHI).
Viziano et al. (36) Italy	Undescribed	47 patients Unilateral vestibular hypofunction.	*N* = 23 Head-mounted display (20 min/day for 1 month) + Conventional VRT {(Adaptation, substitution, habituation, and balance exercises)(30–45 min/times, twice a week for 1 month)}	*N* = 24 Conventional VRT (Adaptation, substitution, habituation, and balance exercises). 30–45 min/times, twice a week for 1 month.	Immersive head-mounted display.	Undescribed.	Vertigo: Dizziness Handicap Inventory (DHI).
Stankiewicz et al. (37) Poland	49.7 ± 10.17/48.2 ± 11.55	20 Patients Unilateral vestibular hypofunction.	*N* = 10 Head-mounted display (2 sessions of 5 min with 5-min intervals for 5 consecutive days) + Conventional VRT {(Cawthorne–Cooksey exercise)(1 time/day for 5 days)}.	*N* = 10 Conventional VRT (Cawthorne–Cooksey exercise). 1 time/day for 5 days.	Immersive head-mounted display (Based on the Google Cardboard platform).	VR Roller Coaster: An entertaining application with an open-top train ride through steep hills and valleys, accompanied by a very strong sensation of gravity shifts.	Vertigo: Vertigo Symptom Scale-Short Form (VSS-SF).
Shu et al. (38) China	53.07 ± 11.12/51.93 ± 11.25	76 patients Primary BPPV.	*N* = 38 Immersion Vestibular Function Rehabilitation Training System. 15 to 20 min/times, 2 times/day for 4 weeks.	*N* = 38 Conventional VRT (Cawthorne–Cooksey exercise). 15 to 20 min/times, 2 times/day for 4 weeks.	Head-mounted display, joystick, immersive vestibular function rehabilitation training system.	1. Introductory level (15 scenarios); 2 weeks of seated practice. 2. Consolidation level (21 scenes); 2 weeks of practice in the standing position.	Vertigo: Dizziness Handicap Inventory (DHI).
Lima Rebêlo et al. (39) Brazil	69.25 ± 5.67/71.41 ± 5.94	37 patients Older adults with balance disorders and risk of falls.	*N* = 20 Head-mounted display for balance training. 2 times/week for 8 weeks.	*N* = 17 Conventional VRT (Balance training in a straight line, around obstacles, gait/balance training on balance platforms and foam mats. Training the front of the trunk for stability). 2 times/week for 8 weeks.	Immersive head-mounted display: Oculus Rift (Consumer Edition, Facebook, United States).	BoxVR: Avoid obstacles by jumping, crouching, and alternately hitting flying circles Bask head: Basketball hoop strapped to the head, with angular movement of the head and torso while shooting a basketball InCell: The head tilts to avoid obstacles when moving forward automatically Thrills and Chills Roller Coasters: Staying upright while the roller coaster is moving (visual stimulation).	Vertigo: Dizziness Handicap Inventory (DHI).
Kanyılmaz et al. (40) Turkey	70 ± 6/70 ± 5	32 patients Elderly patients with dizziness.	*N* = 16 Head-mounted display + Conventional VRT (Eye movements exercise, Gaze stabilization exercises, Postural stability exercises). 30 min/day, 5 times/week, for 3 weeks.	*N* = 16 Conventional VRT (Eye movements exercise, Gaze stabilization exercises, Postural stability exercises). 30 min/day, 5 times/week, for 3 weeks.	Immersive head-mounted display. VR glasses (Samsung Gear VR-SM323), and a smartphone (Samsung Galaxy S7), 360 camera (Samsung Gear 360), and 3D viewing of 15-min and 1.5-min videos.	1. A wide plaza, crowded with pedestrian and automobile traffic, recording realistic ambient noise. 2. An aisle room in a large supermarket, with all the shelves filled with goods of different shapes and colors.	Vertigo: Vertigo Symptom Scale (VSS), Dizziness Handicap Inventory (DHI).
Hasimova et al. (41) Turkey	46.2 ± 12.7/45.3 ± 11.6	87 patients Unilateral vestibular hypofunction.	*N* = 45 Non-immersive gaming console (45 min/session, 2 days/week for 8 weeks) + Home exercise program {(Cawthorne–Cooksey exercises) (30 min/time, ≥ 5 days/week)}.	*N* = 42 Home exercise program (Cawthorne–Cooksey exercises). 30 min/time, ≥ 5 days/week.	Non-immersive gaming console: Nintendo Wii.	“Soccer Heading,” “Slalom Ski,” “Penguin Slide,” “Tight Rope Walk,” “Snowboard Slalom,” “Ski Jumping,” “Table Tilt,” and “Balance Bubble.”	Vertigo: Visual Analog Scale (VAS), Dizziness Handicap Inventory (DHI).
Ozdil et al. (42) Turkey	44.08 ± 14.66/47.33 ± 1.39	31 patients BPPV.	*N* = 10 PlayStation VR + VRT (Adaptation exercises, Habituation exercises, Balance exercises, Gait exercises). 45 to 50 min/day, 3 times/week for 8 weeks.	*N* = 10 Canalith Repositioning Maneuver. 8 weeks.	Immersive head-mounted display. VR headset: 3D glasses, VR headset.	Verti-Go Home game: In the tunnel, dodge the obstacles encountered by rotating the patient’s head in different directions.	Vertigo: Visual Analog Scale (VAS).
Yan et al. (43) China	54.87 ± 3.24/54.55 ± 3.67^a^ 55.11 ± 4.26/54.55 ± 3.67^b^	93 patients Residual symptoms of BPPV.	*N* = 31 Head-mounted display + Cawthorne–Cooksey exercise^a^ /Brandt–Daroff exercise^b^. 15–20 min/time, twice a day for 4 weeks.	*N* = 31^a^ Cawthorne–Cooksey exercise. 10 min/time, twice a day for 4 weeks *N* = 31^b^ Brandt–Daroff exercise 5 times for 4 weeks.	Immersive head-mounted display.	15 scenarios in the introductory and adaptation phase and 21 scenarios in the consolidation and improvement phase.	Vertigo: Dizziness Handicap Inventory (DHI).
AL-Omari et al. (44) Jordan	39.6 ± 8.75/40.9 ± 13.02	40 patients PPPD	*N* = 20 Head-mounted display + Optokinetic stimulation (Photodynamic stimulation video) + Conventional VRT (Gaze stabilization exercises, Postural stability exercises) 45–50 min/times, twice a week for 6 weeks.	*N* = 20 Optokinetic stimulation (Photodynamic stimulation video) + Conventional VRT (Gaze stabilization exercises, Postural stability exercises surface) 45–50 min/times, twice a week for 6 weeks.	Immersive head-mounted display: Samsung Gear VR glasses	Photodynamic stimulation video: Visual Vertigo Video, Car Driving Video and 3D Visual Rainbow Candy Video.	Vertigo: Dizziness Handicap Inventory (DHI).

I, Intervention group; C, Control group; VR, Virtual Reality; VRT, Vestibular Rehabilitation Therapy; BPPV, Benign paroxysmal positional vertigo; PPPD, Persistent postural-perceptual dizziness.

^a^Cawthorne-Cooksey exercise group. ^b^Brandt-Daroff exercise group.

Ten studies (33, 34, 36–40, 42–44) compared head-mounted devices to conventional rehabilitation and two studies (35, 41) compared non-immersive Nintendo Wii to conventional VRT.

Regarding the number of VR interventions in the intervention group, we observed an average of 13.3 sessions (13.3 ± 5.25); Ozdil et al. (42) applied the most sessions, a total of 24. The studies by Yan et al. (43) and Micarelli et al. (34) were used the least number of times with a total of 8 times. The range of intervention durations varied from 4 to 16 weeks. The average duration range of each intervention was 26 to 35 min. The average number of interventions per week was 6 (13.3 ± 4.8). On the other hand, in the control group, the mean number of regular VRT sessions was 11.7 (11.7 ± 6.4). The mean range of time per exercise was 34.7 min.

Post-intervention vertigo symptoms were assessed using various scales across all participants. These instruments have demonstrated reliability as measures of vertigo symptoms. Ten studies (33–36, 38–41, 43, 44) employed the Dizziness Handicap Inventory (DHI), of which Hasimova et al. (41) and Kanyılmaz et al. (40) studies also used Visual Analog Scale (VAS), Vertigo Symptom Scale (VSS) respectively. Ozdil et al. (42) and Stankiewicz et al. (37) study used the VAS scale to measure vertigo. However, the utilization of various assessment tools across different studies has introduced complexity to our analysis. To address this challenge, we adhered to the Cochrane Handbook and standardized the data by calculating statistical measures, such as SMD (45), thereby converting the scores from diverse studies into comparable effect sizes. This approach alleviated the influence of measurement tool discrepancies, facilitating comparisons and analyses of data from various studies within a cohesive framework. Furthermore, 12 studies (33–44) reported enhanced vertigo outcomes for continuous variables, with one study (39) expressing outcomes using 95% CI and one (40) using interquartile range. Subsequently, we applied the data transformation formula recommended by Chi and Luo to calculate the mean and standard deviation.

### 3.3 Quality evaluation

**Figure 2:**
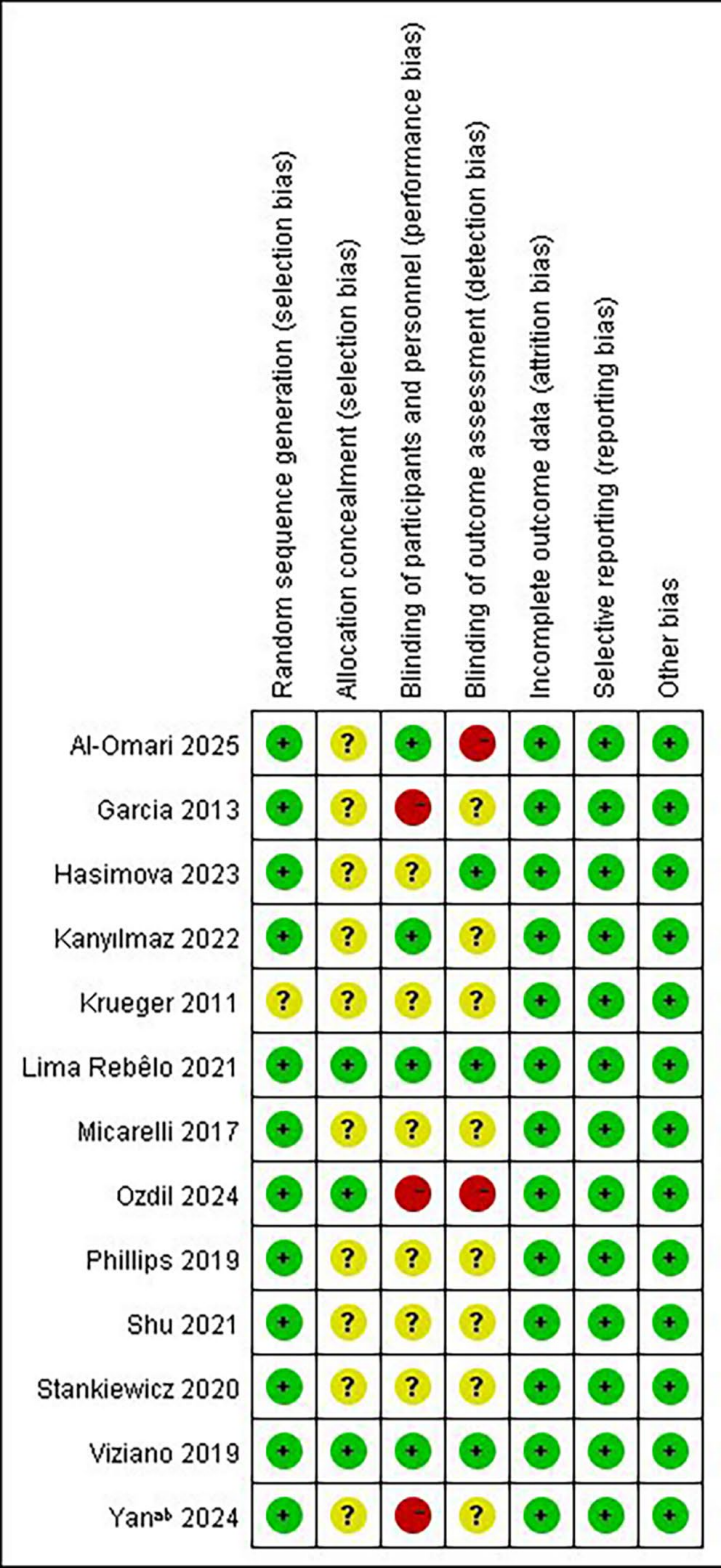
Risk of bias summary.

The results of the risk of bias assessment for the RCTs are shown in Figure 2. 12 studies (33–44) discussed random sequence generation methods, including random number table methods and computerized random sequence methods. The studies of Viziano et al. (36), Lima Rebêlo et al. (39), and Ozdil et al. (42) reported detailed methods of assigning concealment, whereas the other studies were considered as unclear risks. It is worth noting that only the studies by Viziano et al. (36) and Lima Rebêlo et al. (39) were described as double-blind, and the studies by Kanyılmaz et al. (40) and AL-Omari (44) et al. as single-blind. The studies by Garcia et al. (33), Ozdil et al. (42), and Yan et al. (43) explicitly stated that they did not blind patients, and one of them, Ozdil et al. (42), did not blind the evaluators either, and therefore assessed the risk of bias as “high risk,” while the remaining studies were assessed as “unclear risk of implementation bias.” Ten studies (33, 34, 36–39, 41–44) had complete outcome data, and the studies by Philips et al. (35) and Kanyılmaz et al. (40) were rated as “low risk” with reference to the number of reasons and excluded participants.

### 3.4 Meta-analysis results

In total, 12 studies (33–44) examined the effectiveness of VR (immersive or non-immersive) in the rehabilitation of dizziness for patients with PVD. Two studies (35, 41) compared non-immersive VR with conventional VRT, whereas 10 studies (33, 34, 36–40, 42–44) compared immersive VR with conventional VRT. The study by Hasimova et al. (41) used the DHI, VAS scale, and since the study explicitly stated that it was using VAS scale to evaluate vertigo severity, the VAS scale was chosen for variable inclusion. Although the study by Kanyılmaz et al. (40) also used DHI and VSS scales, the VSS scale was mainly used to evaluate the severity of vertigo, so the VSS scale was chosen for inclusion as a variable. The studies yielded continuous variables, assessed by different scales. Thus, SMD was employed as the effect size indicator.

Subsequently, we categorized the studies into two groups based on immersion levels, specifically the types of devices used—immersive and non-immersive VR—and conducted a subgroup analysis focusing on the primary variable of this review: improvement in dizziness.

**Figure 3:**
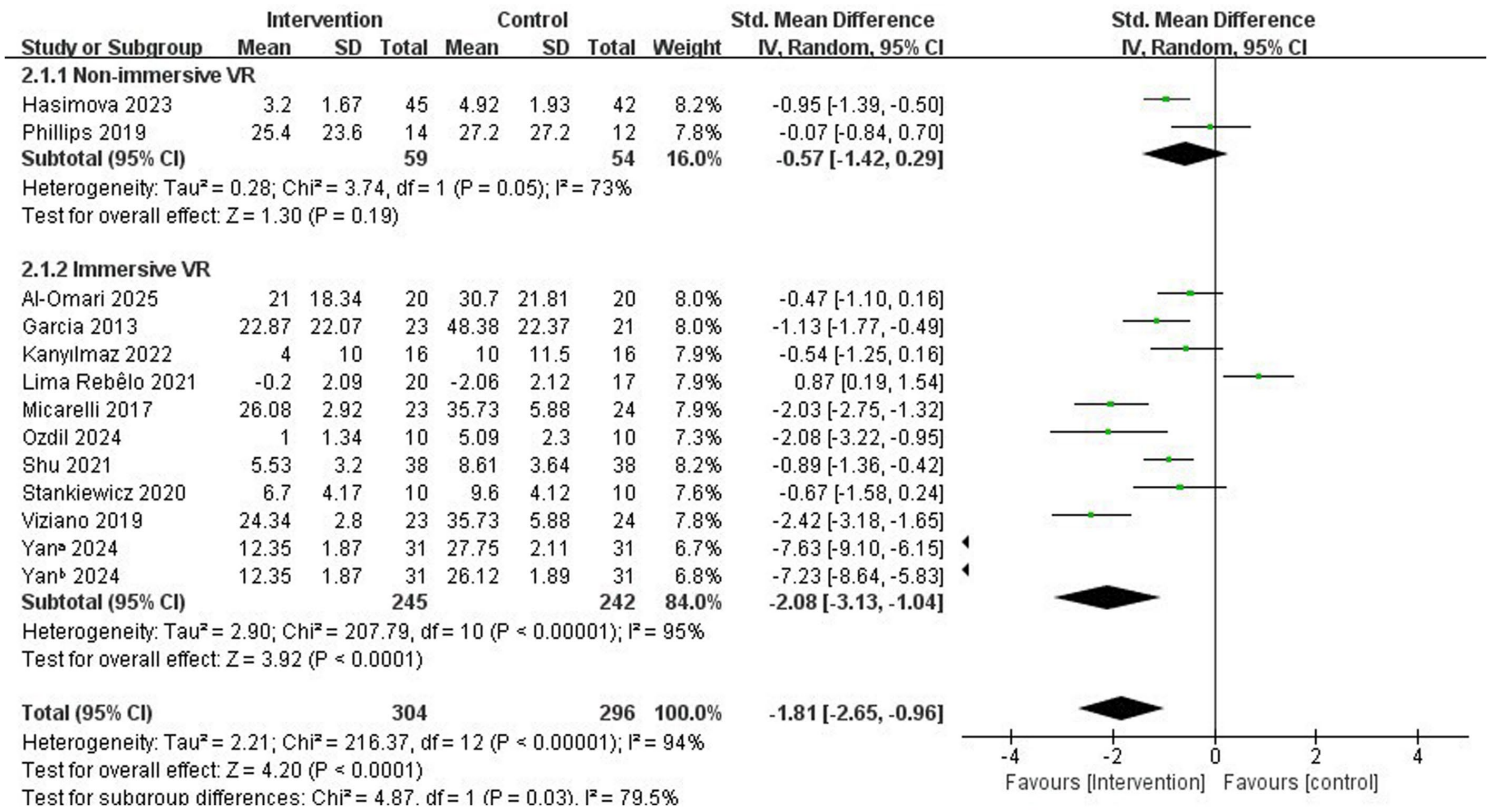
Subgroup analysis of immersive VR: Immersion level.

In subgroup analyses examining immersion level, both non-immersive VR and immersive VR significantly improved vertigo symptoms in patients with PVD. In the specific study focusing on immersive VR, we concluded that vertigo symptoms significantly improved (SMD = −2.08, 95% CI = −3.13 to −1.04, *p* < 0.001; Figure 3).

Given the observed effectiveness of immersive VR interventions in the subgroup analysis, along with the significant heterogeneity (*I^2^* = 95%, *p* < 0.001), this may be attributed to small sample sizes or inherent differences in intervention protocols and study designs among the included studies. We further investigated three intervention parameters as potential sources of heterogeneity: intervention period, single intervention duration, and intervention frequency. These analyses aim to systematically evaluate how VR intervention plans affect the effectiveness of immersive VR.

**Figure 4:**
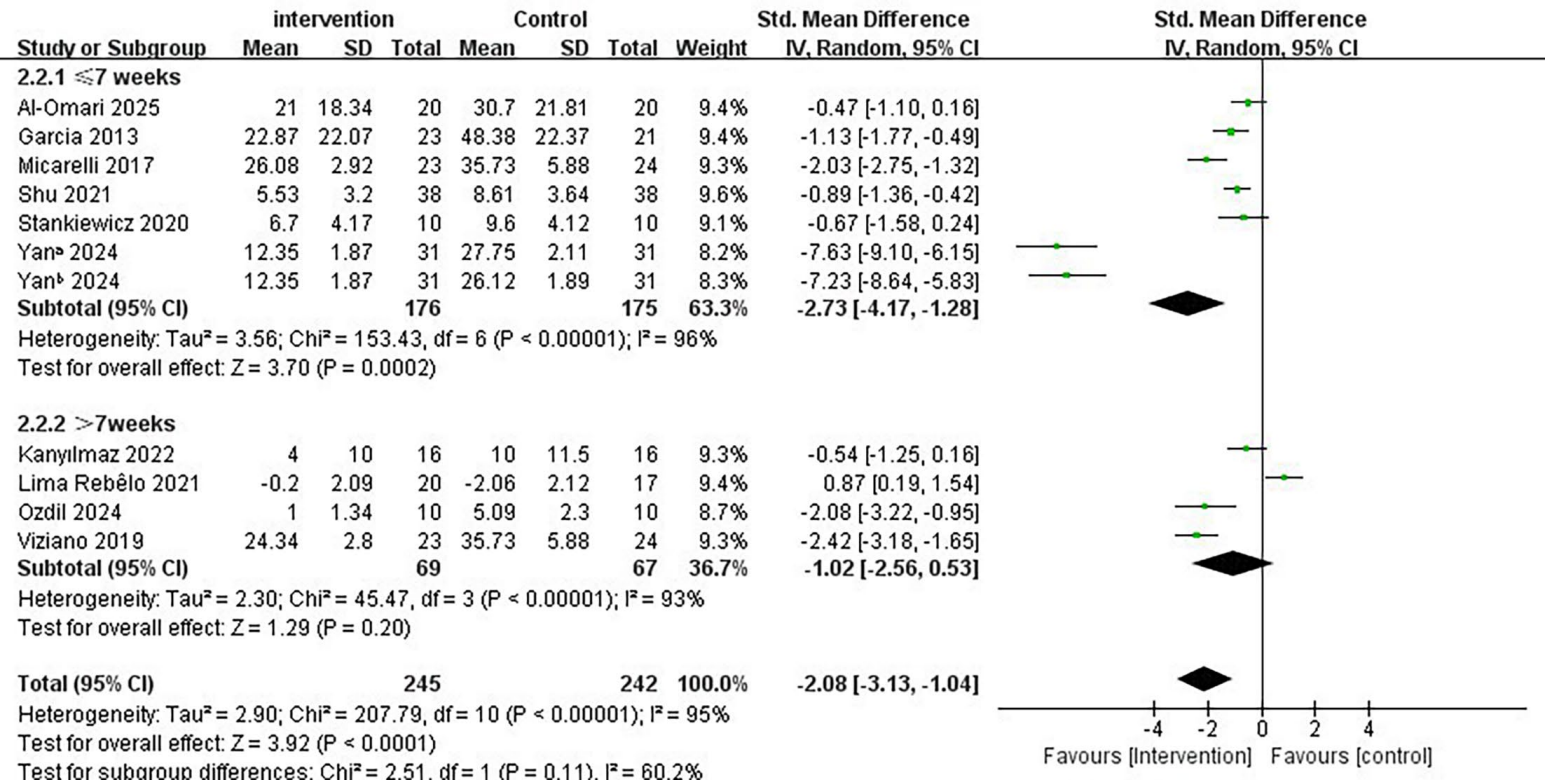
Subgroup analysis of immersive VR: Intervention period ≤7 weeks vs.>7 weeks.

**Figure 5:**
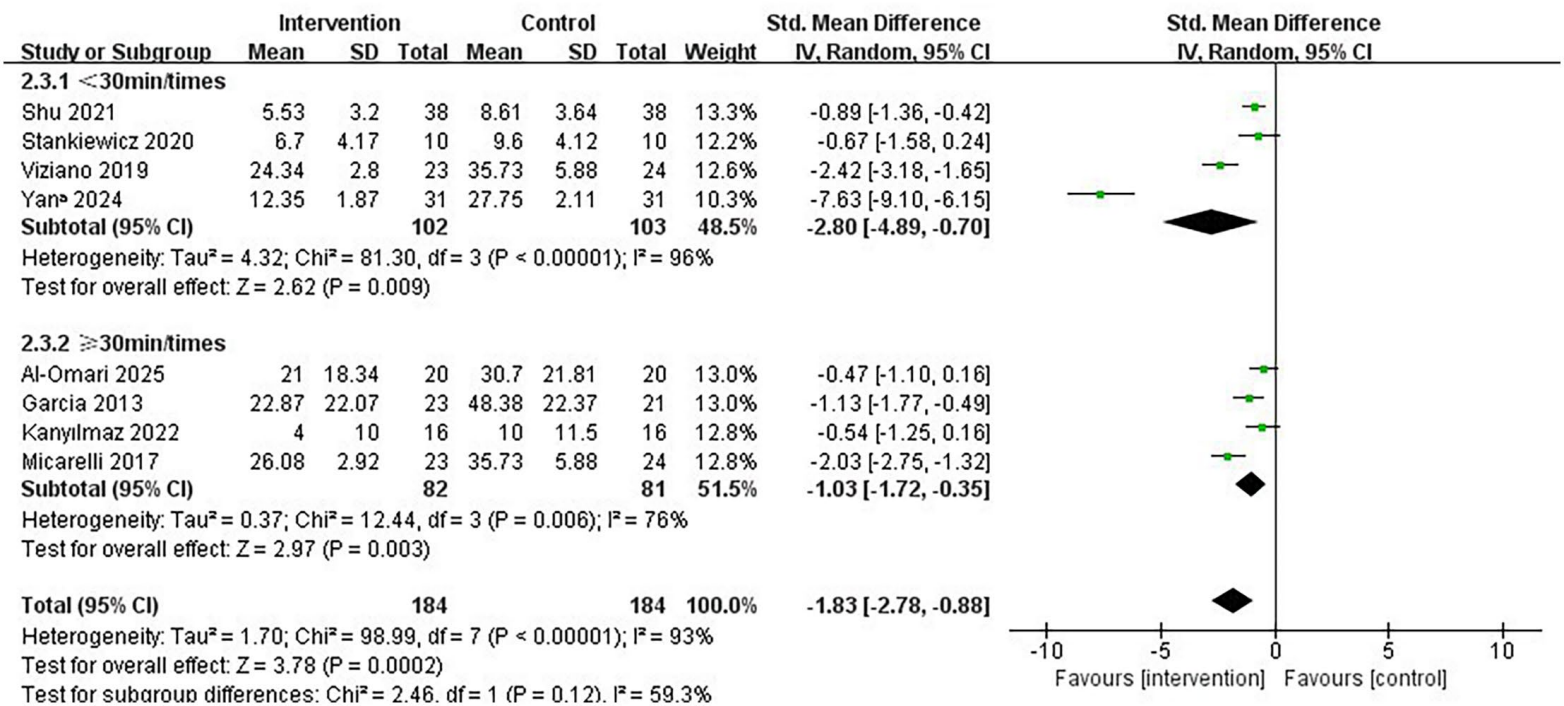
Subgroup analysis of immersive VR: Single intervention time<30 min/times vs. ≥ 30 min/times.

**Figure 6:**
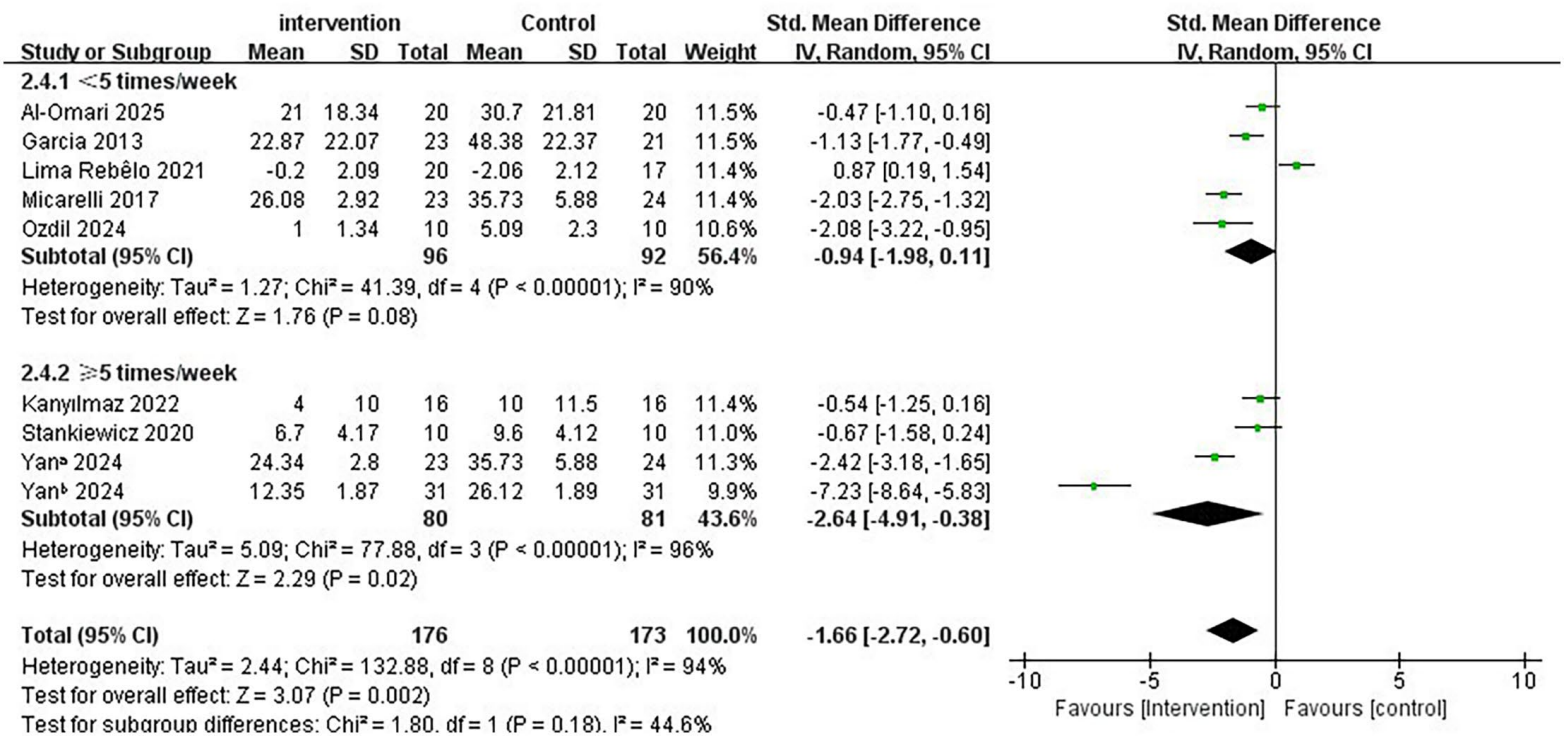
Subgroup analysis of immersive VR: Intervention frequency<5 times/week vs. ≥ 5 times/week.

Regarding the intervention period, a duration of ≤7 weeks significantly improved vertigo symptoms in patients with PVD compared to a duration of >7 weeks (SMD = −2.73; 95% CI = −4.17 to −1.28, *p* < 0.001; Figure 4). Conversely, the longer intervention period >7 weeks showed no statistically significant improvement in vertigo symptoms (SMD = −1.02; 95% CI = −2.56 to 0.53, *p* = 0.20). In terms of single intervention duration, the synthesized effect size of the subgroup with <30 min/times (SMD = −2.80, 95% CI = −4.89 to −0.70, *p* = 0.009; Figure 5) exhibited a slightly larger effect than the subgroup with ≥30 min/times (SMD = −1.03, 95% CI = −1.72 to −0.35, *p* = 0.003). In terms of intervention frequency, intervention frequency ≥5 times/week was effective in improving vertigo symptoms in patients with PVD compared with intervention frequency <5 times/week (SMD = −2.64; 95% CI = −4.91 to −0.38, *p* = 0.02; Figure 6), and there was no statistical significance for intervention frequency <5 times/week (SMD = −1.98, 95% CI = −1.98 to 0.11, *p* = 0.08).

### 3.5 Publication bias and sensitivity analysis

**Figure 7:**
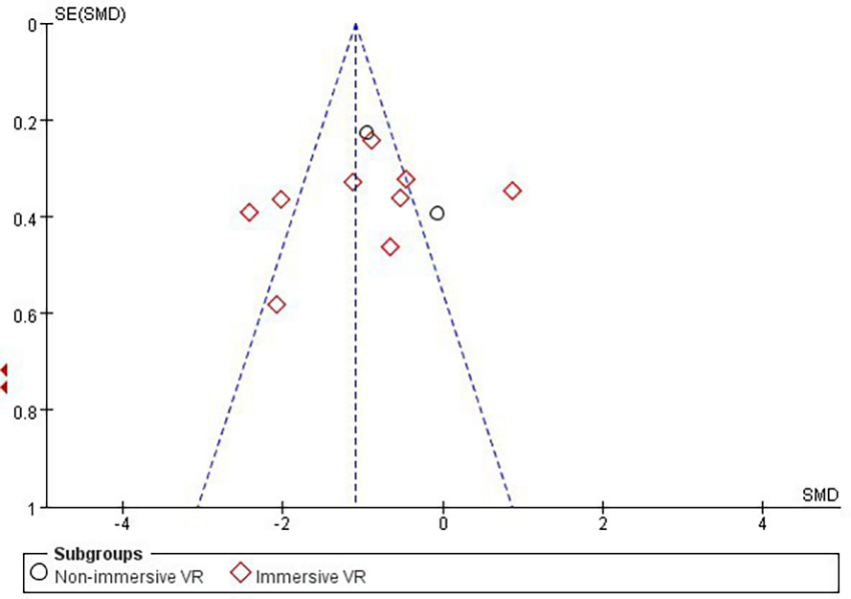
Funnel plot of immersion level.

**Figure 8:**
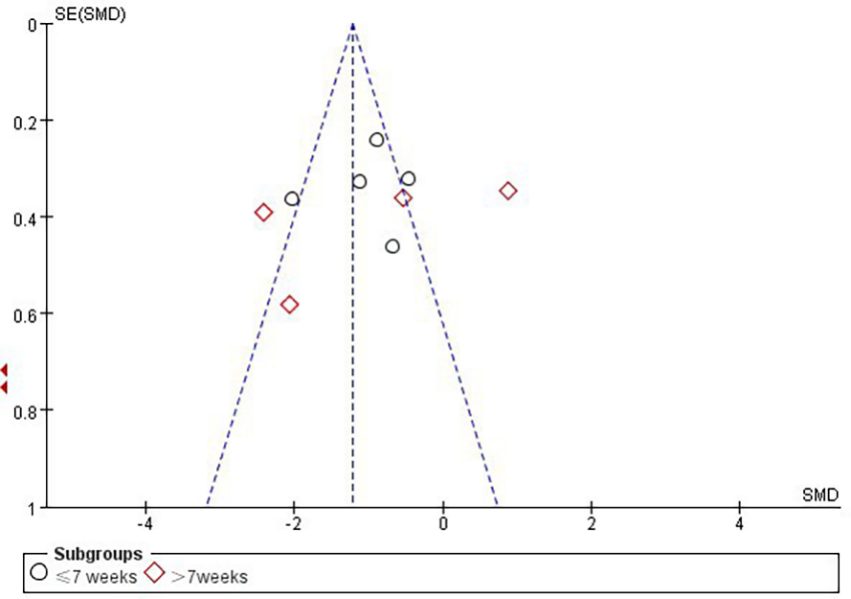
Funnel plot of immersive VR group regarding intervention period studies.

According to Cochrane’s recommendations (30), the use of funnel plots to test for publication bias is more reliable when the number of studies included in the Meta-analysis reaches at least 10. Therefore, the present study maps funnels for immersion levels, as well as for studies involving Intervention period in the immersive VR group. The results showed that the funnel plots presented a roughly left–right symmetrical distribution, as shown in Figures 7, 8. The Egger’s test showed that there was no publication bias against the immersion level (*p* > 0.05). For the studies involving Intervention period in the immersion VR group, there was publication bias (*p* < 0.05). So the cut-and-patch method was adopted to deal with bias, no literature was added, and the data did not change after the cut-and-patch, thus it can be determined that the impact of bias on the results is small. Furthermore, we performed sensitivity analyses on the outcome indicators for the two groups independently. The results indicated that the effect sizes across the studies exhibited minimal change, suggesting that the findings of the meta-analysis are relatively stable.

## 4 Discussion

### 4.1 Summary of findings

The main objective of this study was to investigate the effectiveness of different immersion levels of VR in improving vertigo symptoms in patients with PVD. The systematic review and meta-analysis herein comprehensively analyzed data from 12 RCTs to draw research conclusions. And further review identifies a diverse, generalized range of ideal options for conducting vestibular rehabilitation training. Overall, the use of both immersive and non-immersive VR appeared to help with vestibular rehabilitation, but immersive VR was more effective. Subgroup analyses also found that immersive VR programs with an intervention period of ≤7 weeks, a single intervention duration of <30 min, and an intervention frequency of ≥5 times/week were more effective in improving vertigo.

Our meta-analysis showed that immersive VR showed statistically significant superiority over non-immersive VR and traditional VRT, although the results of this were limited by significant statistical heterogeneity. Patients with PVD utilized VR devices for balance and gait training, enhancing the stability of vestibulo-ocular reflex (46), which in turn stimulated central nervous system compensatory mechanisms and helped in alleviating or eliminating vertigo symptoms (47, 48). This constitutes one of the potential explanations for the systematic review finding that immersive VR is superior to both non-immersive and conventional VRT in mitigating vertigo symptoms, particularly through multisensory stimulation that enhances proprioceptive function (49). Moreover, immersive VR may reduce anxiety and spatial fear during the rehabilitation process, which increasing patients’ confidence in treatment and their motivation to recover (50, 51). Hazzaa’s review corroborates our findings, indicating that immersive VR is a promising approach for effectively improving vertigo symptoms (20). Conversely, Lima Rebêlo’s study (39) reported no significant differences between immersive VR and conventional VRT. This may be due to the fact that the patients included in that study were older, less receptive to VR equipment, and had difficulty concentrating during the training session, ultimately hindering their ability to complete the sessions. Furthermore, the subgroup conducted in this investigation were based on a small number of studies. Importantly, the results of this study are derived from fewer than 1,000 patients and are based on *post hoc* analyses; thus, a large volume of methodologically rigorous studies is necessary to revalidate these results before endorsing immersive VR for widespread clinical application.

The design and specific technical parameters of immersive VR training scenarios are critical for their clinical applications and subsequent research. Kanyılmaz employed two types of immersive 3D VR scenarios: (1) a 15-min outdoor plaza video, captured with a Samsung Gear 360 camera, featuring pedestrians, vehicle traffic, and environmental sounds, in which patients engaged in seated and standing training within a clinical environment; (2) a 1.5-min video of a supermarket shelf filled with colorful products, where patients walked on a treadmill while wearing Samsung Gear VR goggles connected to a Galaxy S7 phone (40). This setup allowed patients to alter their perspective to view products of varying shapes and colors while hearing authentic environmental sounds, thus helping their brains to mitigate dizziness induced by sensory information misalignment through the integration of inputs from multiple senses. Micarelli utilized VR equipment embedded in a ‘Revelation’ 3D VR headset (dimensions: 140 × 105 × 64 mm; weight: 165 g), integrated with a Windows Phone (5.2-inch display, Lumia 930, Windows 10 Mobile, Microsoft Corporation) (34). Patients were required to adjust the eye-tracking device’s settings according to their pupillary distance and focal length prior to each use. Similarly, Lee employed a VR device comprising an Android smartphone (5.8-inch display, Samsung Galaxy S9) placed inside a ‘BOBO VR Z4’ headset (dimensions: 194 × 117 × 127 mm; weight: 410 g), where patients were instructed to focus on a blue ball set against backgrounds of varying difficulty levels while maintaining a consistent speed during 15 head movements (52). However, the studies included in the review did not report essential parameters such as resolution and refresh rate. Subsequently, our review of relevant research revealed that certain high-end VR devices have achieved a resolution of 4 K per eye, with refresh rates reaching 120 Hz or higher and field of view angles extending up to 200° (53, 54). It is worth noting that the weight of VR devices also serves as a critical parameter (55). Given that patients with peripheral vestibular vertigo are predominantly middle-aged and elderly, the prolonged use of heavier VR devices may compress the cervical spine and diminish comfort (56). However, existing studies have reported limited relevant technical parameter indicators. It is expected that high-quality RCTs will be conducted in the future to establish standardized technical parameter indicators. Additionally, by integrating game levels and immersive life scenarios, VR environments enhance the enjoyment and motivation for vestibular training. It is recommended that rehabilitation programs for middle-aged and elderly individuals incorporate personalized setting options within the system, allowing patients to adjust device parameters (e.g., movement speed, visual intensity) according to their individual physical conditions.

Subgroup analyses found that intervention period of ≤7 weeks and single interventions lasting <30 min significantly improved vertigo symptoms in patients with PVD, which is consistent with the vestibular training program recommended by the guidelines (11). This phenomenon may be attributed to the fact that a shorter intervention period not only reduces vestibular adaptation fatigue but also alleviates vertigo symptoms, thereby enhancing patients’ confidence in treatment adherence and the overall effectiveness of vestibular rehabilitation (37, 38). Furthermore, we found that a frequency of ≥5 times/week was more effective on improving vertigo symptoms (37, 40). It may be due to the fact that frequent interventions in a short period of time help to accelerate and strengthen the adaptive and compensatory mechanisms of the vestibular system and improve patient compliance, ultimately improving vertigo symptoms. It is important to note that fully immersive VR also presents certain challenges. During the treatment process, patients with PVD are required to wear head-mounted displays, which can lead to an overload of visual information. Consequently, some participants may experience motion sickness, resulting in dizziness, nausea, and vomiting, thereby diminishing the effectiveness of the intervention (57). This underscores the need for healthcare professionals to assess vestibular function continuously during rehabilitation, allow for flexible adjustments to the training program, and gradually increase training difficulty to achieve optimal intervention outcomes. Furthermore, the variability in the intervention effects among PVD patients, attributable to individual differences such as age and vertigo symptoms, suggests that comprehensive vestibular function assessments should be performed before designing a training program for these patients. This implies that before developing training programs for patients with PVD, a thorough assessment of vestibular function should be conducted, and individualized rehabilitation programs should be formulated, taking into account age, medical history, and cognitive level.

### 4.2 Limitation

The study presents several limitations. First, the sample size included in the study is relatively small, and the nature of VR devices means that not all studies employ blinded methods. This significantly contributes to the Hawthorne effect and potential biases introduced by researchers, which may lead to an overestimation of the efficacy of VR in alleviating dizziness. Second, the study’s results demonstrate substantial heterogeneity. Although sensitivity and subgroup analyses suggest that the meta-analysis results are relatively stable, the precise sources of heterogeneity remain undetermined. This may stem from the lack of uniformity in the tools used for measuring dizziness. Different studies utilize various tools, resulting in discrepancies in dimensions, assessments, and sensitivity. Such variations introduce data collection bias and exacerbate the heterogeneity of the results. Furthermore, the complexity of patients with PVD is another crucial factor. The variability in their causes, severity, and disease course may lead to clinical heterogeneity between studies, thereby challenging the stability of the results. Third, the outcome measures included in this research are subjective, which may introduce additional heterogeneity. It is recommended that future studies incorporate objective measures for assessing dizziness symptoms, such as the Mini-Balance Evaluation Systems Test (Mini-BES Test) and the Modified Clinical Test of Sensory Interaction for Balance (mCTSIB). Finally, most of the studies are single-center designs, and the technical parameters and performance of the VR devices differ, which may impose limitations that could affect treatment outcomes.

## 5 Conclusion

In conclusion, most of the included studies indicate that immersive VR was more effective in improving vertigo symptoms in patients with PVD, where the intervention period was ≤7 weeks, the single intervention time was <30 min, and the intervention frequency was ≥5 times/week were more desirable for the improvement of vertigo symptoms in patients with PVD. It is recommended to establish standardized VR device parameter indicators in the future, and use the same outcome assessment tools as much as possible to conduct larger-sample, multicenter, high-quality studies aimed at providing clear evidence regarding the efficacy of immersive VR for PVD patients. Additionally, the development of scientific and personalized vestibular training programs, along with high-performance VR devices tailored to address the individual needs of elderly patients with PVD, is essential. Providing these patients with personalized vestibular rehabilitation programs and high-quality rehabilitative care services will help achieve the best possible rehabilitation outcomes.

## Data Availability

The original contributions presented in the study are included in the article/Supplementary material, further inquiries can be directed to the corresponding author.

## References

[1] Obermann M Gebauer A Arweiler-Harbeck D Lang S Seilheimer B Kleinschnitz C . Cognitive deficits in patients with peripheral vestibular dysfunction. Eur J Neurol. (2025) 32:e15907. doi: 10.1111/ene.15907, PMID: 37272216 PMC11618113

[2] Nocini R Monzani D Arietti V Giacomo P Segato E Cornale N . Vestibular rehabilitation in adults: an overview. Hear Balance Commun. (2024) 22:31–6. doi: 10.4103/HBC.HBC_6_24

[3] Strupp M Bisdorff A Furman J Hornibrook J Jahn K Maire R . Acute unilateral vestibulopathy/vestibular neuritis: diagnostic criteria. J Vestib Res. (2022) 32:389–406. doi: 10.3233/VES-220201, PMID: 35723133 PMC9661346

[4] Xing Y Si L Zhang W Wang Y Li K Yang X. Etiologic distribution of dizziness/vertigo in a neurological outpatient clinic according to the criteria of the international classification of vestibular disorders: a single-center study. J Neurol. (2024) 271:2446–57. doi: 10.1007/s00415-023-12166-3, PMID: 38231268 PMC11055744

[5] Li X Wei C Gao X Sun J Yang J. Global trends in the research on older population dizziness/vertigo: a 20-year bibliometric and visualization analysis. Braz J Otorhinolaryngol. (2024) 90:101441. doi: 10.1016/j.bjorl.2024.101441, PMID: 38834014 PMC11178979

[6] Ravi K Sivaranjani M Komathi R Sharanya M. Comparing therapeutic maneuvers in posterior canal benign paroxysmal positional vertigo. Indian J Otol. (2023) 29:61. doi: 10.4103/indianjotol.indianjotol_180_22

[7] Hackenberg B O’Brien K Döge J Lackner KJ Beutel ME Münzel T . Vertigo and its burden of disease—results from a population-based cohort study. Laryngoscope Investig Otolaryngol. (2023) 8:1624–30. doi: 10.1002/lio2.1169 PMC10731510 38130247

[8] Pauwels S Lemkens N Lemmens W Meijer K Meyns P Berg RVD . The importance of frailty in older adults with benign paroxysmal positioning vertigo. J Neurol Phys Ther. (2025) 49:99–107. doi: 10.1097/NPT.0000000000000495, PMID: 39805124

[9] Marmor S Karaca-Mandic P Adams ME. Use of physical therapy and subsequent falls among patients with dizziness in the US. JAMA Otolaryngol Head Neck Surg. (2023) 149:1083–90. doi: 10.1001/jamaoto.2023.2840, PMID: 37707824 PMC10502691

[10] Lin ME Gallagher TJ Straughan A Marmor S Adams ME Choi JS. Association of symptomatic dizziness with all-cause and cause-specific mortality. JAMA Otolaryngol Head Neck Surg. (2024) 150:257–64. doi: 10.1001/jamaoto.2023.4554, PMID: 38329761 PMC10853869

[11] Hall CD Herdman SJ Whitney SL Anson ER Carender WJ Hoppes CW . Vestibular rehabilitation for peripheral vestibular hypofunction: an updated clinical practice guideline from the academy of neurologic physical therapy of the American physical therapy association. J Neurol Phys Ther. (2022) 46:118–77. doi: 10.1097/NPT.0000000000000382, PMID: 34864777 PMC8920012

[12] Takeda N Matsuda K Fukuda J Sato G Uno A Kitahara T. Vestibular compensation: neural mechanisms and clinical implications for the treatment of vertigo. Auris Nasus Larynx. (2024) 51:328–36. doi: 10.1016/j.anl.2023.11.009, PMID: 38114342

[13] Alyahya D. A systematic review of effectiveness of vestibular rehabilitation on improving balance in patients with different conditions. Int J Theor Phys. (2022) 9:72–9. doi: 10.15621/ijphy/2022/v9i2/1236

[14] Hall CD Flynn S Clendaniel RA Roberts DC Stressman KD Pu W . Remote assessment and management of patients with dizziness: development, validation, and feasibility of a gamified vestibular rehabilitation therapy platform. Front Neurol. (2024) 15:1367582. doi: 10.3389/fneur.2024.1367582, PMID: 38872821 PMC11169667

[15] Law JH Koh HY Kua A. Optokinetic stimulation in the rehabilitation of visually induced dizziness in people with vestibular disorders: a systematic review. Clin Rehabil. (2024) 38:1001–22. doi: 10.1177/02692155241244932, PMID: 38584422

[16] Andreev A Mindova S. Advancements in vestibular physiotherapy: a comprehensive review. J IMAB. (2024) 30:5829–33. doi: 10.5272/jimab.2024304.5829

[17] Pan S Hu Y Zhang H He Y Tian C Lei J. The current status and trends of research related to vestibular disorders, vertigo, and cognitive impairment in the elderly population: a bibliometric analysis. Ear Nose Throat J. (2024) 16:1455613241257396. doi: 10.1177/01455613241257396, PMID: 38818829

[18] Maggio MG Cezar RP Milardi D Borzelli D De Marchis C D’Avella A . Do patients with neurological disorders benefit from immersive virtual reality? A scoping review on the emerging use of the computer-assisted rehabilitation environment. Eur J Phys Rehabil Med. (2024) 60:37–43. doi: 10.23736/S1973-9087.23.08025-5 37971719 PMC10939039

[19] Swaminathan B Shanmugam VU Shanmugam R Prabhash PR Siddiqi M Divya PS. 3D virtual reality rehabilitation therapy for patients with vertigo due to peripheral vestibular dysfunction. Indian J Otolaryngol Head Neck Surg. (2023) 75:2222–6. doi: 10.1007/s12070-023-03678-5, PMID: 37636637 PMC10447306

[20] Hazzaa NM Manzour AF Yahia E Mohamed Galal E. Effectiveness of virtual reality-based programs as vestibular rehabilitative therapy in peripheral vestibular dysfunction: a meta-analysis. Eur Arch Otorrinolaringol. (2023) 280:3075–86. doi: 10.1007/s00405-023-07911-3, PMID: 36947249 PMC10220119

[21] Quan W Liu S Cao M Zhao J. A comprehensive review of virtual reality technology for cognitive rehabilitation in patients with neurological conditions. Appl Sci. (2024) 14:6285. doi: 10.3390/app14146285

[22] Thorp SO Rimol LM Lervik S Evensmoen HR Grassini S. Comparative analysis of spatial ability in immersive and non-immersive virtual reality: the role of sense of presence, simulation sickness and cognitive load. Front Virtual Real. (2024) 5:3872. doi: 10.3389/frvir.2024.1343872

[23] Comparcini D Simonetti V Galli F Saltarella I Altamura C Tomietto M . Immersive and non-immersive virtual reality for pain and anxiety management in pediatric patients with hematological or solid cancer: a systematic review. Cancers. (2023) 15:985. doi: 10.3390/cancers15030985, PMID: 36765945 PMC9913167

[24] Sokolowska B Swiderski W Smolis-Bak E Sokolowska E Sadura-Sieklucka T. A machine learning approach to evaluate the impact of virtual balance/cognitive training on fall risk in older women. Front Comput Neurosci. (2024) 18:1390208. doi: 10.3389/fncom.2024.1390208, PMID: 38808222 PMC11130377

[25] García-Sánchez M García-Robles P Osuna-Pérez MC Lomas-Vega R Obrero-Gaitán E Cortés-Pérez I. Effectiveness of virtual reality-based early postoperative rehabilitation after total knee arthroplasty: a systematic review with meta-analysis of randomized controlled trials. Appl Sci. (2023) 13:4597. doi: 10.3390/app13074597

[26] Park M Cho Y Na G Kim J. Application of virtual avatar using motion capture in immersive virtual environment. Int J Hum-comput Interact. (2024) 40:6344–58. doi: 10.1080/10447318.2023.2254609

[27] Heffernan A Abdelmalek M Nunez DA. Virtual and augmented reality in the vestibular rehabilitation of peripheral vestibular disorders: systematic review and meta-analysis. Sci Rep. (2021) 11:17843. doi: 10.1038/s41598-021-97370-9, PMID: 34497323 PMC8426502

[28] Le Perf G Thebault G Duflos C Herman F Cauquil-Gleizes S Laffont I. Can virtual reality replace conventional vestibular rehabilitation tools in multisensory balance exercises for vestibular disorders? A non-inferiority study. J Neuroeng Rehabil. (2025) 22:86. doi: 10.1186/s12984-025-01623-x, PMID: 40253374 PMC12008832

[29] Tugwell P Tovey D. Prisma 2020. J Clin Epidemiol. (2021) 134:A5–6. doi: 10.1016/j.jclinepi.2021.04.008 34637928

[30] Higgins JPT Altman DG Gøtzsche PC Jüni P Moher D Oxman AD . The cochrane collaboration’s tool for assessing risk of bias in randomised trials. BMJ. (2011) 343:d5928. doi: 10.1136/bmj.d5928, PMID: 22008217 PMC3196245

[31] Chi K-Y Li M-Y Chen C Kang E Taiwan C. Ten circumstances and solutions for finding the sample mean and standard deviation for meta-analysis. Syst Rev. (2023) 12:62. doi: 10.1186/s13643-023-02217-1 37005690 PMC10068165

[32] Luo D Wan X Liu J Tong T. Optimally estimating the sample mean from the sample size, median, mid-range, and/or mid-quartile range. Stat Methods Med Res. (2018) 27:1785–805. doi: 10.1177/0962280216669183, PMID: 27683581

[33] Garcia AP Ganança MM Cusin FS Tomaz A Ganança FF Caovilla HH. Vestibular rehabilitation with virtual reality in ménière’s disease. Braz J Otorhinolaryngol. (2013) 79:366–74. doi: 10.5935/1808-8694.20130064, PMID: 23743754 PMC9443828

[34] Micarelli A Viziano A Augimeri I Micarelli D Alessandrini M. Three-dimensional head-mounted gaming task procedure maximizes effects of vestibular rehabilitation in unilateral vestibular hypofunction: a randomized controlled pilot trial. Int J Rehabil Res. (2017) 40:325–32. doi: 10.1097/MRR.0000000000000244, PMID: 28723718

[35] Phillips JS Fitzgerald J Phillis D Underwood A Nunney I Bath A. Vestibular rehabilitation using video gaming in adults with dizziness: a pilot study. J Laryngol Otol. (2018) 132:202–6. doi: 10.1017/S0022215118000075, PMID: 29512476

[36] Viziano A Micarelli A Augimeri I Micarelli D Alessandrini M. Long-term effects of vestibular rehabilitation and head-mounted gaming task procedure in unilateral vestibular hypofunction: a 12-month follow-up of a randomized controlled trial. Clin Rehabil. (2019) 33:24–33. doi: 10.1177/0269215518788598, PMID: 30012022

[37] Stankiewicz T Gujski M Niedzielski A Chmielik LP. Virtual reality vestibular rehabilitation in 20 patients with vertigo due to peripheral vestibular dysfunction. Med Sci Monit. (2020) 26:e930182. doi: 10.12659/MSM.930182, PMID: 33543735 PMC7871733

[38] Shu F Shi L Zhang Q Hui L. Curative effect of the immersive vestibular function rehabilitation training system on residual symptoms after successful canalith repositioning maneuvers in patients of BPPV. J Audiol Speech Pathol. (2021) 29:509–13. doi: 10.3969/j.issn.1006-7299.2021.05.008

[39] Lima Rebêlo F de Souza Silva LF Doná F Sales Barreto A de Souza Siqueira Quintans J. Immersive virtual reality is effective in the rehabilitation of older adults with balance disorders: a randomized clinical trial. Exp Gerontol. (2021) 149:111308. doi: 10.1016/j.exger.2021.111308, PMID: 33744393

[40] Kanyılmaz T Topuz O Ardıç FN Alkan H Öztekin SNS Topuz B . Effectiveness of conventional versus virtual reality-based vestibular rehabilitation exercises in elderly patients with dizziness: a randomized controlled study with 6-month follow-up. Braz J Otorhinolaryngol. (2022) 88 Suppl 3:S41–9. doi: 10.1016/j.bjorl.2021.08.010, PMID: 34799265 PMC9760985

[41] Hasimova Z Sahbaz T Karacay BC Karan A. Effectiveness of virtual reality therapy in chronic unilateral vestibular hypofunction: a randomized controlled study. Turk J Phys Med Rehabil. (2023) 69:286–93. doi: 10.5606/tftrd.2023.12360, PMID: 37674792 PMC10478541

[42] Ozdil A Iyigun G Balci B. Three-dimensional exergaming conjunction with vestibular rehabilitation in individuals with benign paroxysmal positional vertigo: a feasibility randomized controlled study. Medicine. (2024) 103:e38739. doi: 10.1097/MD.0000000000038739, PMID: 38968532 PMC11224863

[43] Yan S Gao P Wu W. Role of comprehensive vestibular rehabilitation based on virtual reality technology in residual symptoms after canalith repositioning procedure. J Int Adv Otol. (2024) 20:272–8. doi: 10.5152/iao.2024.231393, PMID: 39128125 PMC11232068

[44] Al-Omari MM Abuzaid SM Khair HJ Manaf H Alghwiri AA. The effect of using virtual reality on balance in people with persistent postural-perceptual dizziness: a randomized controlled trial. J Vestib Res. (2025) 35:213–24. doi: 10.1177/09574271251326587, PMID: 40085770

[45] Higgins JPT Chandler J Cumpston M Li T Page MJ Welch VA. Cochrane handbook for systematic reviews of interventions version 6.5. London: Cochrane (2024).

[46] Warchoł J Tetych A Tomaszewski R Kowalczyk B Olchowik G. Virtual reality-induced modification of vestibulo-ocular reflex gain in posturography tests. J Clin Med. (2024) 13:2742. doi: 10.3390/jcm13102742, PMID: 38792284 PMC11122614

[47] Kalron A Frid L Fonkatz I Menascu S Dolev M Magalashvili D . The design, development, and testing of a virtual reality device for upper limb training in people with multiple sclerosis: single-center feasibility study. JMIR Serious Games. (2022) 10:e36288. doi: 10.2196/36288, PMID: 36094809 PMC9513692

[48] Lilios A Nikitas C Skoulakis C Alagianni A Chatziioannou I Asimakopoulou P . The unveiled potential of telehealth practice in vestibular rehabilitation: a comparative randomized study. J Clin Med. (2024) 13:7015. doi: 10.3390/jcm13237015, PMID: 39685471 PMC11642665

[49] Peng K Moussavi Z Karunakaran KD Borsook D Lesage F Nguyen DK. iVR-fNIRS: studying brain functions in a fully immersive virtual environment. Neurophotonics. (2024) 11:20601. doi: 10.1117/1.NPh.11.2.020601, PMID: 38577629 PMC10993907

[50] Bu X Ng PHF Xu W Cheng Q Chen PQ Cheng ASK . The effectiveness of virtual reality-based interventions in rehabilitation management of breast cancer survivors: systematic review and meta-analysis. JMIR Serious Games. (2022) 10:e31395. doi: 10.2196/31395, PMID: 35225817 PMC8922144

[51] Bell IH Pot-Kolder R Rizzo A Rus-Calafell M Cardi V Cella M . Advances in the use of virtual reality to treat mental health conditions. Nat Rev Psychol. (2024) 3:552–67. doi: 10.1038/s44159-024-00334-9

[52] Lee JW Yoon CY Kim JH Seo YJ Kong TH. Virtual reality-based vestibular rehabilitation therapy in patients with acute unilateral vestibulopathy: a randomized controlled trial. Front Neurol. (2025) 16:1519470. doi: 10.3389/fneur.2025.1519470, PMID: 39935612 PMC11810741

[53] Park J-H Lee B. Holographic techniques for augmented reality and virtual reality near-eye displays. Light-adv Manuf. (2022) 3:9. doi: 10.37188/lam.2022.009

[54] Wong ES Wahab NHA Saeed F Alharbi N. 360-degree video bandwidth reduction: technique and approaches comprehensive review. Appl Sci. (2022) 12:7581. doi: 10.3390/app12157581

[55] Yazdipour AB Saeedi S Bostan H Masoorian H Sajjadi H Ghazisaeedi M. Opportunities and challenges of virtual reality-based interventions for patients with breast cancer: a systematic review. BMC Med Inform Decis Mak. (2023) 23:17. doi: 10.1186/s12911-023-02108-4, PMID: 36691014 PMC9872398

[56] Kupczik L Farrelly W Wilson S. Appraising virtual technologies’ impact on older citizens’ mental health-a comparative between 360° video and virtual reality. Int J Environ Res Public Health. (2022) 19:11250. doi: 10.3390/ijerph191811250, PMID: 36141517 PMC9517141

[57] Park S Lee G. Full-immersion virtual reality: adverse effects related to static balance. Neurosci Lett. (2020) 733:134974. doi: 10.1016/j.neulet.2020.134974, PMID: 32294492

