# Supplementary material for: Comparison of the effectiveness of immersive and non-immersive virtual reality in the treatment of vertigo in patients with peripheral vestibular dysfunction: a systematic review and meta-analysis
Source: Front Neurol. 2025 Jul 17;16:1638868. doi: 10.3389/fneur.2025.1638868 (PMC12312637; doi:10.3389/fneur.2025.1638868)
Supplement: Supplementary file 1 [file Data_Sheet_1.docx]

**PubMed 512**

#1 (((((((((((((((((peripheral vertigo[MeSH Terms]) OR (peripheral vertigo[Title/Abstract])) OR ((peripheral[Title/Abstract]) AND (vertigo[Title/Abstract]))) OR (meniere disease[MeSH Terms])) OR (Meniere's disease[Title/Abstract])) OR (Meniere's disease)) OR (Benign Paroxysmal Positional Vertigo)) OR (Benign Paroxysmal Positional Vertigo[Title/Abstract])) OR (Vestibular Neuritis[Title/Abstract])) OR (Vestibular Neuritis)) OR (Vestibular Neuritis[MeSH Terms])) OR (BPPV[Title/Abstract])) OR (BPPV)) OR (Peripheral vestibular dysfunction)) OR (Peripheral vestibular dysfunction[Title/Abstract])) OR (Peripheral vestibular lesion[Title/Abstract])) OR (vertigo*[Title/Abstract]))

#2 (((((((((((virtual reality[MeSH Terms]) OR ((virtual[Title/Abstract]) AND (reality[Title/Abstract]))) OR (virtual reality[Title/Abstract])) OR (virtual environment[Title/Abstract])) OR (digital technology[Title/Abstract])) OR (video game[Title/Abstract])) OR (exergaming[Title/Abstract])) OR (virtual rehabilitation[Title/Abstract])) OR (VR[Title/Abstract])) OR (Three-dimensional[Title/Abstract])) OR (3D Virtual Reality[Title/Abstract])))

#3 #1 AND #2

**MEDLINE 482**

#1 ((((((((((MH=(vertigo) OR ((TS=(peripheral)) AND TS=(vertigo)))) OR TS=(peripheral vertigo)) OR MH=(meniere disease)) OR TS=(Meniere's disease)) OR TS=(Benign Paroxysmal Positional Vertigo)) OR TS=(Vestibular Neuritis)) OR MH=(Vestibular Neuritis)) OR TS=(BPPV)) OR TS=(Peripheral vestibular dysfunction)) OR TS=(Peripheral vestibular lesion)

#2 ((((((((((MH=(virtual reality) OR (TS=(virtual)) AND TS=(reality)) ) OR TS=(virtual reality)) OR TS=(virtual environment)) OR TS=(digital technology)) OR TS=(video game)) OR TS=(exergaming)) OR TS=(virtual rehabilitation)) OR TS=(Three-dimensional)) OR TS=(VR)) OR TS=(3D Virtual Reality)

#3 #1 AND #2

**Web of Science 297**

#1 (((((((((((TS= (peripheral vertigo) OR ((TS=(peripheral)) AND TS=(vertigo))) OR TS=(Meniere's disease)) OR ALL=(Meniere's disease)) OR TS=(Benign Paroxysmal Positional Vertigo)) OR ALL=(Benign Paroxysmal Positional Vertigo)) OR TS=(Vestibular Neuritis)) OR ALL=(Vestibular Neuritis)) OR TS=(BPPV)) OR ALL=(BPPV)) OR TS=(Peripheral vestibular dysfunction)) OR ALL=(Peripheral vestibular dysfunction)) OR TS=(Peripheral vestibular lesion)

#2 ((((((((TS= (virtual reality) OR ((TS=(virtual)) AND TS=(reality))) OR TS=(virtual environment)) OR TS=(digital technology)) OR TS=(video game)) OR TS=(exergaming)) OR TS=(virtual rehabilitation)) OR TS=(VR)) OR TS=(Three-dimensional)) OR TS=(3D Virtual Reality)

#3 #1 AND #2

**Embase 337**

#1 'peripheral vertigo'/exp OR 'peripheral origin vertigo':ab,ti OR 'peripheral vestibular vertigo':ab,ti OR 'vertigo of peripheral nervous origin':ab,ti OR 'vertigo of peripheral origin':ab,ti OR 'peripheral vertigo':ab,ti OR 'meniere disease'/exp OR 'meniere disease':ab,ti OR 'meniere disease' OR 'benign paroxysmal positional vertigo' OR 'vestibular neuronitis':ab,ti OR 'vestibular neuronitis' OR 'vestibular neuronitis'/exp OR 'benign paroxysmal positional vertigo':ab,ti OR 'peripheral vestibular dysfunction' OR 'peripheral vestibular dysfunction':ab,ti OR 'peripheral vestibular lesion':ab,ti

#2 'virtual reality'/exp OR 'virtual reality':ab,ti OR 'virtual reality' OR 'virtual environment':ab,ti OR 'digital technology':ab,ti OR 'video game':ab,ti OR 'exergaming':ab,ti OR vr:ab,ti OR 'three dimensional':ab,ti OR '3d virtual reality':ab,ti

#3 #1 AND #2

**CINAHL 248**

(SU (virtual reality OR digital technology) OR TX (virtual reality OR virtual environment OR digital technology OR video game OR exergaming OR virtual rehabilitation OR Three-dimensional OR 'VR' OR 3D Virtual Reality)) AND (SU (peripheral vertigo OR Meniere's disease OR Benign Paroxysmal Positional Vertigo OR Vestibular Neuritis OR BPPV OR Peripheral vestibular dysfunction OR Peripheral vestibular lesion) OR (Benign Paroxysmal Positional Vertigo OR Vestibular Neuritis OR BPPV OR Peripheral vestibular dysfunction))

**Cochrane Library 87**

#1 MeSH descriptor: [Vertigo] explode all trees

#2 MeSH descriptor: [Meniere Disease] explode all trees

#3 MeSH descriptor: [Vestibular Neuronitis] explode all trees

#4 (peripheral vertigo):ti,ab,kw OR (Meniere's disease OR Benign Paroxysmal Positional Vertigo OR Vestibular Neuritis OR BPPV OR Peripheral vestibular dysfunction OR Peripheral vestibular lesion):ti,ab,kw OR (Benign Paroxysmal Positional Vertigo OR Vestibular Neuritis OR BPPV OR Peripheral vestibular dysfunction) (Word variations have been searched) 1452

#5 #1 OR #2 OR #3 OR #4

#6 MeSH descriptor: [Virtual Reality] explode all trees

#7 (virtual reality OR virtual environment OR digital technology OR video game OR exergaming OR virtual rehabilitation OR Three-dimensional OR 'VR' OR 3D Virtual Reality):ti,ab,kw (Word variations have been searched)

#8 #6 OR #7

#9 #5 AND #8

**WanFang：327**

主题:(虚拟现实 OR 虚拟环境 OR 虚拟游戏 OR 沉浸式 OR 交互式 OR “VR” OR 数字技术) and 主题:(眩晕 OR 梅尼埃病 OR 良性阵发性位置性眩晕 OR 前庭神经炎 OR 耳石症 OR BPPV OR 前庭功能 OR 晕动症)

**CNKI：128**

(主题:虚拟现实 +虚拟环境 + 虛拟游戏 +沉浸式 + 交互式+VR + 数字技术)AND(主题:眩晕 +梅尼埃病 +良性阵发性位置性眩晕 +前庭神经炎 +耳石症 + BPPV + 前庭功能 + 晕动症)

**VIP：197**

任意字段：:(虚拟现实 OR 虚拟环境 OR 虚拟游戏 OR 沉浸式 OR 交互式 OR “VR” OR 数字技术) and 任意字段:(眩晕 OR 梅尼埃病 OR 良性阵发性位置性眩晕 OR 前庭神经炎 OR 耳石症 OR BPPV OR 前庭功能 OR 晕动症)

**CBM：49**

(( "眩晕"[常用字段:智能] OR "梅尼埃病"[常用字段:智能] OR "良性阵发性位置性眩晕"[常用字段:智能] OR "前庭神经炎"[常用字段:智能] OR "耳石症"[常用字段:智能] OR "BPPV"[常用字段:智能] OR "前庭功能"[常用字段:智能] OR "晕动症"[常用字段:智能])) AND (( "虚拟现实"[常用字段:智能] OR "虚拟环境"[常用字段:智能] OR "虚拟游戏"[常用字段:智能] OR "沉浸式"[常用字段:智能] OR "交互式"[常用字段:智能] OR "VR"[常用字段:智能] OR "数字技术"[常用字段:智能]))
